# Janus kinase inhibitors for alopecia areata: a review of clinical data

**DOI:** 10.3389/fimmu.2025.1577115

**Published:** 2025-05-13

**Authors:** Yutong Sun, Qian Li, Yanli Zhang, Yaling Liu

**Affiliations:** Department of Dermatology, Hebei Medical University Third Hospital, Shijiazhuang, China

**Keywords:** alopecia areata, Janus kinases, baricitinib, ritlecitinib, deuruxolitinib

## Abstract

Alopecia areata (AA) is an autoimmune disease characterized by inflammatory and non-scarring hair loss, mediated by CD8+ T cells and primarily affecting hair follicles. Janus kinase (JAK) inhibitors selectively inhibit JAK, block the signal transducer and activator of transcription pathway, and often interfere with T-cell-mediated inflammatory cytokine pathways. They are a class of targeted anti-inflammatory drugs that can promote the activation of hair follicle stem cells. Studies have shown that JAK inhibitors exhibited good efficacy and safety in the treatment of AA, with fewer serious side effects. This article reviews the mechanism of action of JAK inhibitors in the treatment of AA and the effects and side effects of representative drugs.

## 1 Introduction

Although alopecia areata (AA) is not life threatening, it does affect physical appearance, psychology, and daily life, as it can recur or even progress in most patients. From 1990 to 2021, global AA incidence has increased in absolute terms (1). The lifetime prevalence of AA is 0.7%–3.8%, and 7%–12% of patients progress to total or general alopecia (2). The estimated lifetime prevalence of AA in the overall population is 0.10% worldwide, with 0.12% of adults and 0.03% of children affected (3).

**Figure 1:**
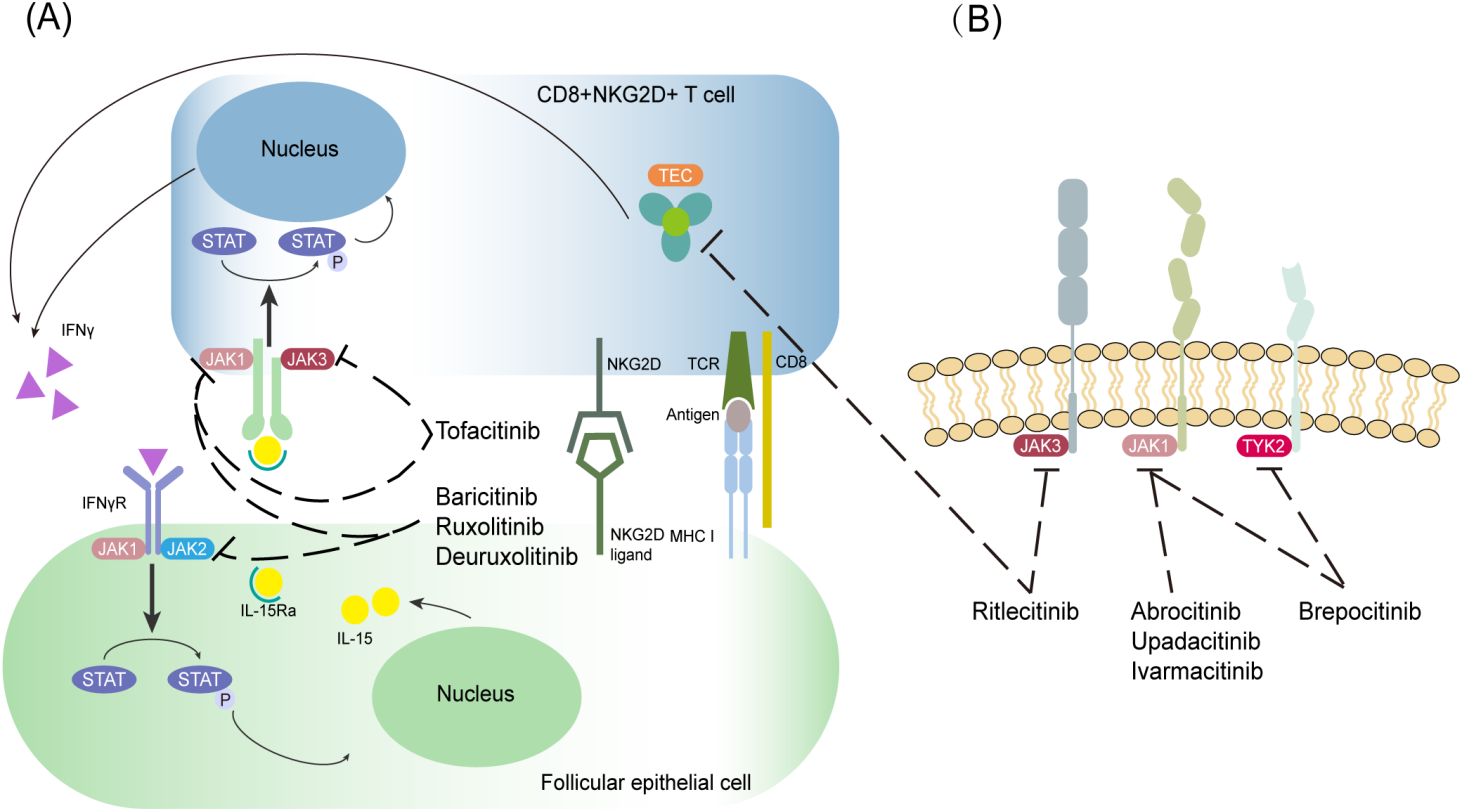
Schematic diagram of the mechanism of JAK inhibitors for the treatment of AA. **(A, B)** JAK-STAT-dependent immune pathway in AA. The targets of various JAK inhibitors are indicated in the figure, and the black dashed line with a blunt tip indicates inhibition.

AA is caused by a number of factors, and the common theory is the breakdown of the immune privilege of the hair follicle. Hair follicle atrophy and hair loss occur when follicular stem cells, dermal papilla cells, and related proteins undergo progressive damage via CD8+ T-cell-mediated inflammatory pathways and cytokine cascades. This pathological process mainly involves immunological recognition abnormalities, inflammatory amplification signaling, and microenvironmental dysregulation within the hair follicle. As a type of special CD8+ T cell, cytotoxic CD8+NKG2D+ T cells carry the NKG2D receptor (product of the *KLRK1* gene), which is one of the natural killer immune receptors (4, 5). Stimulatory factors in patients with AA produce interferon-gamma (IFN-γ) through a positive feedback loop between follicular epithelial and CD8+NKG2D+ T cells via the Janus kinase (JAK)-signal transducer activator of transcription (STAT) pathway, which promotes the loss of follicular immune privilege (6). In patients with AA, hair loss results from T-cell-induced inflammation in the hair follicle regions, which disrupts the growth cycle and impairs its function (7, 8). Lymphocytic infiltration and several pro-inflammatory cytokines, such as interleukin (IL)-15 and IFN-γ, also contribute to hair loss (9, 10). As shown in **Figure 1**, JAK inhibitors specifically inhibit JAK1/2 in hair follicle epithelial cells and JAK1/3 in T cells, thereby inhibiting CD8+NKG2D+ T-cell activity and STAT phosphorylation and reducing IL-15 and IFN-γ secretion to slow the loss of hair follicle immune privilege. This pathway is initiated when follicular epithelial cells present self-antigens to CD8+NKG2D+ T cells via major histocompatibility complex I antigen and NKG2D/NKG2D ligand complexes and activate CD8+NKG2D+ T cells. Activated CD8+NKG2D+ T cells produce IFN-γ, which binds to the corresponding IFN-γ receptor on follicular epithelial cells, triggering the downstream JAK1/2-STAT pathway. This leads to the upregulation of IL-15 and IL-15R α, which in turn binds to activated CD8+NKG2D+ T cells and triggers further upregulation of IFN-γ through the JAK1/3-STAT pathway. These molecular interactions create a positive feedback loop between the two cell types to amplify the inflammatory immune response.

Moreover, genetic factors (11), hypersensitivity (12–14), and gut microorganisms (15) are also strongly associated with the development of AA. Immune-related genes, such as MBL-associated serine protease 2, toll-like receptor 1, and chromosome 11 open reading frame 30, are significantly associated with susceptibility to AA. Alternatively, patients with atopic AA exhibiting elevated IgE and IL-4 levels and mast cells/eosinophils count in lesions may benefit from adjunctive allergy therapy to enhance standard AA treatment efficacy (13). Furthermore, gut microbiomes, including *Parabacteroides distasonis* and *Clostridiales vadin* BB60, are associated with alopecia universalis (16). AA cannot be cured immediately; its treatment mainly includes drug, physical, and surgical treatments, such as laser and microneedling, which are less effective for severe and special AA types. Since 2014, scientists have been using JAK inhibitors to treat AA, atopic dermatitis (AD), and other chronic inflammatory diseases, and they have shown promising efficacy (5). Targeted small molecule inhibitors are more precise, with fewer side effects than conventional immunological drugs. In this article, we reviewed the research progress on JAK inhibitors in AA treatment.

## 2 Research progress on JAK inhibitors in AA treatment

Currently, the first-generation JAK inhibitors, such as tofacitinib, baricitinib, and ruxolitinib, are mostly the non-selective types, with a wide inhibition but poor selectivity. Second-generation JAK inhibitors are more precise, with a narrow inhibition window but good selectivity: ritlecitinib and abrocitinib specifically inhibit JAK3 and JAK1, as shown in **Table 1**.

**Table 1:** JAK inhibitor-related therapy.

JAKi	Target	Dosages and Effects
Tofacitinib	JAK1 JAK3	Adults: 58% of 77 patients (5–10 mg bid) achieved>50% improvement in SALT score over 4–18 months of oral treatment (22). Adolescents aged 12–17 years: nine of 13 patients (5 mg bid) experienced clinically significant hair regrowth. The median percent change in SALT score was 93% at an average of 6.5 months of oral treatment (23). Children aged 8–10 years: three of four patients (5 mg qid to 5 mg bid) achieving >60% regrowth at 6 months of oral treatment (24). Children aged ≤5 years: three patients (2.5–5 mg qid) showed >50%, >90%, and >50% improvements at 6, 12, and 21 months, respectively (25). Topical: eight of 11 patients (4–16 years old) had some response to topical 2% tofacitinib, and three of these had cosmetically acceptable regrowth (26).
Baricitinib	JAK1 JAK2	Adult: of the patients in the BRAVE-AA1 (N = 465) and BRAVE-AA2 (N = 390) treated with baricitinib 4 and 2 mg, respectively, 40.9% and 21.2% in BRAVE-AA1 and 36.8% and 24.4% in BRAVE-AA2 achieved a SALT score ≤20 at Week 52 (27). Adolescents aged 12–17 years: 23 of 29 patients treated with baricitinib 4 mg daily during 10 months experienced partial or complete scalp hair regrowth, with a median percentage change in SALT score of 93% (30). Children aged ≤12 years: five of 22 children achieved complete regrowth (SALT 0%), and 10 out of 22 achieved a SALT score of <20% when treated with baricitinib 4 mg for at least 24 weeks (29).
Ruxolitinib	JAK1 JAK2	Nine of 12 patients with moderate-to-severe alopecia areata demonstrated an average hair regrowth of 92% at the end of 3–6 months of treatment (33). Two patients experienced sustained, near-complete regrowth after 1 year of treatment (34). Five of eight patients with severe AA (≥50% scalp hair loss) achieved a mean improvement in SALT score of 98% with 10–25 mg twice daily after nearly 14 months of treatment (35).
Deuruxolitinib	JAK1 JAK2	Percentage of patients achieving a SALT score ≤20 at week 24 in THRIVE AA-1 (706 patients) (39–42): 30% in the 8 mg group, 42% in the 12 mg group, and 1% in the placebo group Percentage of patients achieving a SALT score ≤20 at week 24 in THRIVE AA-2 (517 patients) (39–42):33% in the 8 mg group, 38.3% in the 12 mg group, and 0.8% in the placebo group
Ritlecitinib	JAK3 Tec	Percentage of patients with 30% improvement in SALT score from baseline in ALLEGRO-2a (48 patients) (44): 50% in the ritlecitinib group after 4 weeks (200 mg qid) + 20 weeks (50 mg qid) Percentage of patients achieving a SALT score ≤20 at week 24 in ALLEGRO-2b/3 (718 patients) (45): 31% in the (200 mg+50 mg) group, 22% in the (200 mg+30 mg) group 23% in the 50 mg group, 14% in the 30 mg group, and 2% in the placebo group
Brepocitinib	JAK1 TYK2	Percentage of patients with 30% improvement in SALT score from baseline in ALLEGRO-2a (47 patients) (44): After 4 weeks (60 mg qid) + 20 weeks (30 mg qid), 64% in the brepocitinib group
Abrocitinib	JAK1	46.15%, 53.85%, and 38.46% of patients treated with oral abrocitinib (50–200 mg/d) for at least 3 months achieved SALT score ≤20, 50% of hair regrowth, and 75% of hair regrowth (54).
Upadacitinib	JAK1	The median absolute SALT score of 25 patients (15–30 mg qid) decreased from 50 to 25 at week 12 and then to 5 at week 24 (57).
Ivarmacitinib	JAK1	Least squares mean of percentage change in SALT scores relative to baseline at week 24 in patients with 25%–49% hair loss (59): 19.62% in the 2 mg group, 61.97% in the 4 mg group, 52.51% in the 8 mg, and 28.68% in the placebo group.

### 2.1 Tofacitinib

Tofacitinib selectively inhibits JAK1/JAK3 within the JAK-STAT signaling pathway, blocking IFN-γ receptor-mediated cytokine signaling and suppressing T-cell activation (17, 18). By disrupting the positive feedback loop between CD8+NKG2D+ T and follicular epithelial cells, it reduces IL-15 and IFN-γ secretion, thereby preserving hair follicle immune privilege.

A total of 80 patients treated with tofacitinib for >6 months had a remission rate of 33.8% after 24 weeks of treatment (19). In a retrospective study that included 35 patients with AA, 10 patients (32.3%) achieved almost complete or total scalp hair regrowth (20). The study population primarily involved treatment-refractory patients with advanced alopecia areata, including severe, totalis, and universalis subtypes, with tofacitinib demonstrating therapeutic efficacy for these challenging cases. Based on the retrospective data on 32 long-term Korean patients with moderate to severe AA, Park et al. found that 56.3% of the patients had a 50% improvement in the Severity of Alopecia Tool (SALT) scores from baseline with a daily dose of 10 mg tofacitinib. They found that the duration of tofacitinib (rather than the total dose) was associated with a favorable response (21). In addition, Liu et al. studied the effect of tofacitinib in the treatment of 90 patients with AA, alopecia totalis, or alopecia universalis, with the majority of patients relapsing within 2–3 months of stopping the medication, suggesting that long-term treatment may be required to keep the disease quiescent (22). Adult patients (5–10 mg bid) (22), adolescent patients (5 mg bid) (23), preadolescent children (5 mg qid to 5 mg bid) (24), and children aged ≤5 years (2.5–5 mg qid) (25) had a favorable systemic response to tofacitinib. Colleen et al. found that eight out of 11 pediatric patients responded to topical treatment with 2% tofacitinib, and hair regrowth was cosmetically effective in three of these cases (26).

Although tofacitinib has not been formally approved for AA, accumulating clinical trial evidence and therapeutic experience support its efficacy in patients with AA of different ages and refractory subtypes (1).

### 2.2 Baricitinib

Baricitinib, a reversible JAK1/JAK2 inhibitor, was approved by the US Food and Drug Administration in June 2022 and the Chinese National Drug Administration in March 2023 for the treatment of adults with severe AA.

In one Phase 2 trial and two Phase 3 trials (BRAVE AA-1 and BRAVE AA-2), baricitinib showed an improved efficacy, which continued for over 52 weeks. The two Phase 3 trials enrolled 654 and 546 patients treated with baricitinib 4 and 2 mg, respectively; 40.9% and 21.2% in the BRAVE AA-1 group and 36.8% and 24.4% in the BRAVE AA-2 group achieved a SALT score ≤20 at week 52 (27). Baricitinib is more likely to achieve ≥80% scalp hair coverage compared with placebo, and common adverse events included infection, headache, acne, and elevated blood creatine phosphokinase level (28). In addition, a retrospective longitudinal cohort study was conducted to evaluate the safety and efficacy of baricitinib in the treatment of patients with AA aged <12 years treated for at least 24 weeks, 19 of whom showed a mean reduction in SALT scores of 68% after treatment with a mean daily dose of 4 mg baricitinib (29). A total of 29 adolescent patients (aged 12–17 years) with moderate to severe AA were evaluated, and 23 patients (79%) achieved partial or complete hair regrowth after treatment with baricitinib for at least 3 months with an average final baricitinib dose of 4 mg daily (30). Baricitinib currently appears to have a more favorable risk–benefit ratio compared with available treatment options (particularly systemic immunosuppressants) for the treatment of AA (31, 32).

Clinical trial data indicate that baricitinib demonstrates efficacy and favorable tolerability across diverse age groups with moderate to severe AA. As a promising novel therapeutic agent, additional investigations are necessary to establish long-term safety profiles and efficacy outcomes within this patient population (27, 29–31).

### 2.3 Ruxolitinib and deuruxolitinib

Ruxolitinib specifically inhibits the JAK1/JAK2-related pathway, and although not formally approved for the treatment of AA, there have been a number of successful case studies and reports in recent years.

Ruxolitinib showed efficacy in nine out of 12 patients with moderately severe AA in the high-dose group (20 mg bid) (33), two patients with severe AA (one patient with chronic AA and one patient with acute episodes of AA) in the medium-dose group (30 mg qid) (34), and five out of eight patients with severe AA in the low-dose group (10–25 mg bid) (35). In a double-blind, controlled Phase 2 study, there was no statistically significant difference in the trend of SALT scores between the experimental and control groups after the topical application of ruxolitinib cream (36), suggesting that oral ruxolitinib is effective; however, topical application has no significant effect in patients with AA. To some extent, the relative systemic bioavailability of topical ruxolitinib was significantly lower than that of oral ruxolitinib, possibly due to insufficient penetration. Hair regrowth was not maintained after ruxolitinib was discontinued, and some patients even lost hair. Delaying the end of treatment and tapering the dose may be helpful in maintaining hair growth (34). In addition, the role of ruxolitinib in protecting hair follicles by acting on immune effectors and inhibiting the downstream effects of IFN-γ signaling has been demonstrated by an *in vitro* hair follicle culture model (37).

Recently, deuruxolitinib, a deuterated form of ruxolitinib, has been approved by the US Food and Drug Administration for the treatment of severe AA, following the approval of baricitinib and ritlecitinib for the treatment of AA. Deuterium-substituted drugs are made by replacing one or more carbon–hydrogen (C–H) bonds with carbon–deuterium (C–D) bonds at specific metabolic sites in the drug molecule. This substitution prolongs the half-life of the drug, reduces the production of toxic metabolites and drug–drug interactions, lowers the administered dose, improves safety, and results in improved efficacy. In a double-blind, placebo-controlled Phase 2 study of 149 patients, those treated with 8 mg or 12 mg deuruxolitinib experienced a significant reduction in the severity of AA. One of these patients experienced a serious adverse event of grade 3 cellulitis with sinus congestion, sinus infection, and influenza (38). To further evaluate the efficacy and safety of deuruxolitinib, two large studies involving 1,223 patients, THRIVE AA-1 and THRIVE AA-2, were conducted. In the THRIVE AA-1 study, the proportions of achieving a SALT score of ≤20 in the 8 mg group, 12 mg, and placebo groups were 30%, 42%, and 1%, respectively; in the THRIVE AA-2 study, these proportions were 33%, 38.3%, and 0.8%, respectively. Both doses of deuruxolitinib resulted in significant scalp hair regrowth from 8 weeks. They continued for 24 weeks and were generally well tolerated, with their overall safety profile in patients with AA suggesting their suitability for clinical use (39–42).

### 2.4 Ritlecitinib and brepocitinib

Ritlecitinib is an irreversible, selective JAK3/TEC kinase inhibitor. It selectively inhibits JAK3 by irreversibly covalently binding to the Cys residue at position 909 of the catalytic structural domain. In contrast, in other JAKs, the residue at this position is replaced by a serine. Members of the TEC kinase family have a Cys residue at the same position as JAK3 and are, therefore, also inhibited by ritlecitinib. In the cellular environment, ritlecitinib inhibits the cytolytic activity of natural killer and CD8+ T cells and the production of IFN-γ by inhibiting members of the TEC kinase family (43).

Ritlecitinib was approved by the US Food and Drug Administration in June 2023 and by the Chinese National Drug Administration in October 2023 for the treatment of severe AA in adolescents and adults aged ≥12 years. It is the second JAK inhibitor approved for the treatment of AA after baricitinib and the first approved for the treatment of adolescents with AA. Compared with other JAK inhibitors, ritlecitinib offers a novel mechanism of action with a rapid onset of action and a high safety profile. In the ALLEGRO-2a study, about half of the 48 patients with AA who had more than 50% scalp hair loss showed a 30% improvement in SALT scores from baseline after 24 weeks of oral ritlecitinib, demonstrating good efficacy and safety of ritlectinib (44). In the randomized, double-blind ALLEGRO-2b/3 study, ritlecitinib was well tolerated in 718 adult and adolescent patients with severe AA, with approximately 22–31% achieving a SALT score ≤20 at week 24 (45). Piliang et al. found that hair regrowth was maintained up to week 48 in patients with an initial response at week 24. Approximately one-third of patients who did not initially meet the efficacy target at week 24 achieved a response with continued ritlecitinib treatment (46). Blair et al. analyzed data from selected ALLEGRO-2b/3 and ALLEGRO-LT trials and concluded that ritlecitinib has clinically meaningful and sustained long-term efficacy in patients with AA (47). King et al. reviewed the ALLEGRO trial series and found that ritlecitinib was well tolerated in patients with AA (48). Common adverse reactions included headache, positive new coronavirus tests, nasopharyngitis, acne, and upper respiratory tract infections, and milder symptoms.

Brepocitinib (PF-06700841) selectively inhibits TYK2 and JAK1 channels. Although brepocitinib is not yet approved, studies have shown that it has a significant efficacy. King et al. found that 64% and 50% of patients treated with brepocitinib and ritlecitinib, respectively, had a 30% improvement in their SALT scores compared with baseline (44). Peeva et al. found in the phase 2a ALLEGRO-2a trial of brepocitinib and ritlecitinib that patients who had a poor response to ritlecitinib may benefit from treatment with brepocitinib (49). Guttman et al. found that at week 24, patients in both the brepocitinib and ritlecitinib arms had significant improvements in AA lesion areas that were close to or exceeded the level of non-lesion areas at baseline (50). At week 12, brepocitinib showed greater improvement in scalp hair than ritlecitinib; however, at week 24, ritlecitinib showed more significant improvement.

### 2.5 Abrocitinib and upadacitinib

**Figure 2:**
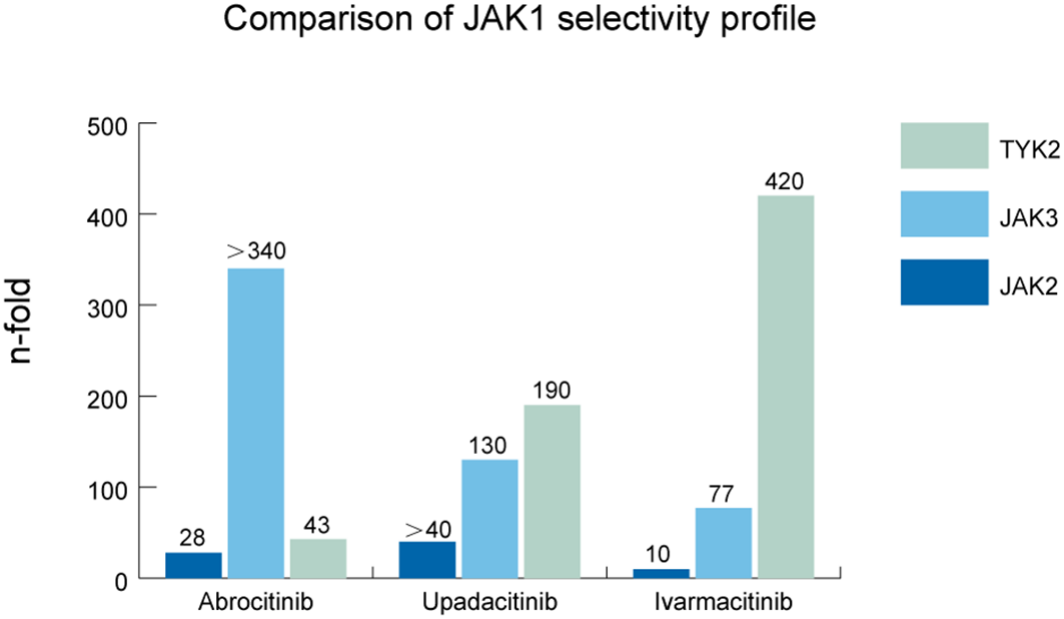
Comparison of JAK1 selectivity profile. Grouped bar graph showing the fold selectivity of JAK1 compared with other JAK subtypes (JAK2, JAK3, and TYK2) for abrocitinib, upadacitinib, and ivarmacitinib in biochemical assays. Numerical labels indicate specific fold differences, with “>40” and “>340” denoting values exceeding the upper detection limit. This figure demonstrates the high specificity of abrocitinib, upadacitinib, and ivarmacitinib for JAK1 inhibition.

As shown in **Figure 2**, abrocitinib showed greater selectivity for JAK1 than JAK2 (28-fold), JAK3 (>340-fold), and TYK2 (43-fold) in biochemical assays (51). One patient with both AD and AA experienced complete regrowth of scalp hair and significant improvement in AD after 1 year of abrocitinib treatment (52). Significant hair regrowth was reported after prolonged abrocitinib treatment in two patients with AD with severe AA (53). In a retrospective study of 13 patients with AA treated with oral abrocitinib (50–200 mg/d) for at least 3 months, 46.15%, 53.85%, and 38.46% of patients achieved SALT score ≤20, 50% of hair regrowth, and 75% of hair regrowth, respectively (54).

Upadacitinib showed greater selectivity for JAK1 than JAK2 (>40-fold), JAK3 (130-fold), and TYK2 (190-fold) in cellular assays and was initially shown to be effective in the treatment of AD (55). Cantelli et al. reported that switching to upadacitinib in a patient with both AD and AA after failures of multiple therapies resulted in a significant improvement in both AD and AA (56). Flora et al. included 25 patients with AA between October 2021 and July 2022 (four patients had a history of AD) and found that the median absolute SALT score decreased from 50 to 25 at week 12 and then to 5 at week 24 (57).

These studies suggest that abrocitinib and upadacitinib may be effective treatment options for patients with both AD and AA.

### 2.6 Ivarmacitinib

Ivarmacitinib (formerly SHR0302), a highly selective JAK1 inhibitor, exhibits potency and selectivity for JAK1 that is over 10-fold for JAK2, 77-fold for JAK3, and 420-fold for TYK2 in cellular assays (58). Zhou et al. set up three doses of 2, 4, and 8 mg/day to test the appropriate dose in a phase 2 clinical trial (59). The results showed dose dependence at the 2 and 4 mg/day doses but not at the 8 mg/day dose. It was hypothesized that the drug may have reached its maximum therapeutic effect at the 4 mg/day dose. At week 24, the least square mean percentage change from baseline in SALT scores was 19.62%, 61.97%, 52.51%, and 28.68% in the 2, 4, 8 mg/day, and placebo groups, respectively, in patients with 25%–49% hair loss. The drug is currently in phase 3 clinical trials in China. Additionally, in a systematic review, baricitinib was likely the most effective therapy, followed by ritlecitinib and ivarmacitinib. However, both of the former showed a dose-dependent effect but not in ivarmacitinib (60).

## 3 Side effects

Systemic JAK inhibitors are well tolerated, with most side effects being mild with a very low discontinuation rate of 1.6% compared with 2.2% in the control group (61). The most common side effect of JAK inhibitors is infection, especially viral (herpes and influenza), fungal, and mycobacterial infectious disorders (62). The most commonly reported serious adverse reactions include varicella-zoster, pneumonia, tuberculosis, sepsis, and non-melanoma skin cancer (63). These characteristics are shown in **Table 2**.

**Table 2:** JAK inhibitor indications and side effects.

JAKi	Indications	Side Effects
Tofacitinib	FDA Approval: Rheumatoid arthritis Psoriatic arthritis Ulcerative colitis	Upper respiratory tract infections, urinary tract infections, headaches Abdominal discomfort, acne, adverse cardiovascular events, and cancer
Baricitinib	FDA Approval: AA Rheumatoid arthritis	Infections, headache, acne, and elevated blood creatine phosphokinase level
Ruxolitinib	FDA Approval: Myelofibrosis, Polycythemia vera Graft-versus-host disease Atopic dermatitis	Upper respiratory infections, weight gain worsening or development of new acne, bruising, and fatigue
Deuruxolitinib	FDA Approval: AA	Upper respiratory tract infections, nasopharyngitis Elevated creatine kinase levels, acne, and headaches
Ritlecitinib	FDA Approved: AA Vitiligo, Ulcerative colitis and Crohn’s disease	Upper respiratory tract infections, nasopharyngitis, acne, and headaches
Brepocitinib	AA Vitiligo, Ulcerative colitis and Crohn's disease	Upper respiratory tract infection, nasopharyngitis, acne, headache, and nausea Severe rhabdomyolysis adverse events without acute kidney injury (2/47)
Abrocitinib	FDA approval: Atopic dermatitis	Nausea, headache, dizziness, nasopharyngitis Symptoms of upper respiratory tract infection, vomiting, thrombocytopenia
Upadacitinib	Rheumatoid arthritis, Psoriatic arthritis Atopic dermatitis, Ankylosing spondylitis	Nausea, headache
Ivarmacitinib	Ulcerative colitis Atopic dermatitis	Upper respiratory tract infection, folliculitis, urinary tract infection, drowsiness, acne

Most first-generation JAK inhibitors are pan-JAK inhibitors with more adverse events such as upper respiratory tract infections, urinary tract infections, headaches, cardiovascular events, and cancer (64). A study comparing tofacitinib with tumor necrosis factor inhibitors showed that tofacitinib was associated with a higher risk of adverse cardiovascular events and cancer in patients with rheumatoid arthritis (65). Patients on JAK inhibitors also often have elevated levels of low-density lipoprotein, a known risk factor for cardiovascular disease, which, in most cases, declines during treatment (63, 66).

Second-generation JAK inhibitors are more selective and have significantly fewer side effects than first-generation JAK inhibitors. The most common adverse events in patients treated with abrocitinib were nasopharyngitis, nausea, and acne (67). Flora et al. found that during the first 24 weeks of upadacitinib treatment, no one experienced significant laboratory abnormalities or infectious complications, and no participants discontinued treatment due to adverse events (57). In a randomized, placebo-controlled phase 2a study, ritlecitinib had the least number of adverse events (82,105, 124) and the lowest proportion of participants experiencing adverse events (67%,74%, and 77%) compared with placebo and brepocitinib, with nasopharyngitis and headache more common (44).

For serious adverse events, ruxolitinib, tofacitinib, and baricitinib were associated with infectious adverse events [IC025 1.7, especially viral infection (herpes and influenza)] and thrombosis (IC025 0.4), tofacitinib was associated with gastrointestinal perforation events (IC025 1.5). There is not a significant increase in the reporting of major cardiovascular events, where adverse events were considered significant if the lower end of the 95% credibility interval of the IC (IC025) was positive (62). However, isolated cases of adverse effects have been reported, including myocardial infarction with baricitinib, hypertensive urgency with tofacitinib, neutropenia with baricitinib, brepocitinib, and deuruxolitinib (61).

There is currently a lack of post-marketing surveillance data, and much attention needs to be paid to individual patient outcomes with JAK inhibitors.

## 4 Conclusion

Compared with the traditional treatments, the effect of JAK inhibitors on severe AA is promising. JAK inhibitors can bring significant improvement to patients who do not respond to corticosteroids or traditional immunosuppressants, patients whose disease has lasted for more than 10 years, and patients whose eyebrows and eyelashes are affected. There is a possibility that JAK inhibitors may be beneficial for patients with AA with other comorbidities such as psoriasis, vitiligo, and AD. In severe AA, oral JAK inhibitors have shown more significant effects, and topical medications that act like intralesional steroids are ideal for children and patients with localized disease because of their limited area of action and ability to minimize the side effects associated with systemic medications. However, there may be a high relapse rate with short-term use or in more severely affected patients; therefore, long-term maintenance therapy may be required to achieve sustained remission. In China, baricitinib and ritlecitinib have been approved for the treatment of AA. Overall, JAK inhibitors have significantly changed the challenging landscape for the treatment of moderate to severe AA, and their efficacy is now widely recognized. Now, more attention needs to be paid to the conditions of use, safety, and tailoring of JAK inhibitors to individual patient characteristics to ensure that they can deliver the best outcomes.

However, it should be noted that the conclusions are limited by the retrospective nature of some of the studies, the small number of patients, and the lack of a control group, and large-scale clinical trials are needed to further validate these effects. The promising use of various JAK inhibitors in the treatment of AA is currently being investigated intensively.
